# Classification of drug-naive children with attention-deficit/hyperactivity disorder from typical development controls using resting-state fMRI and graph theoretical approach

**DOI:** 10.3389/fnhum.2022.948706

**Published:** 2022-08-18

**Authors:** Masoud Rezaei, Hoda Zare, Hamidreza Hakimdavoodi, Shahrokh Nasseri, Paria Hebrani

**Affiliations:** ^1^Department of Medical Physics, Faculty of Medicine, Mashhad University of Medical Sciences, Mashhad, Iran; ^2^Medical Physics Research Center, Mashhad University of Medical Sciences, Mashhad, Iran; ^3^Neuroimaging and Analysis Group, Research Center for Science and Technology in Medicine, Imam Khomeini Hospital, Tehran University of Medical Sciences, Tehran, Iran; ^4^Psychiatry and Behavioral Sciences Research Center, Mashhad University of Medical Sciences, Mashhad, Iran

**Keywords:** attention-deficit/hyperactivity disorder (ADHD), resting-state fMRI, machine learning approach, functional MRI, graph theory

## Abstract

**Background and objectives:**

The study of brain functional connectivity alterations in children with Attention-Deficit/Hyperactivity Disorder (ADHD) has been the subject of considerable investigation, but the biological mechanisms underlying these changes remain poorly understood. Here, we aim to investigate the brain alterations in patients with ADHD and Typical Development (TD) children and accurately classify ADHD children from TD controls using the graph-theoretical measures obtained from resting-state fMRI (rs-fMRI).

**Materials and methods:**

We investigated the performances of rs-fMRI data for classifying drug-naive children with ADHD from TD controls. Fifty six drug-naive ADHD children (average age 11.86 ± 2.21 years; 49 male) and 56 age matched TD controls (average age 11.51 ± 1.77 years, 44 male) were included in this study. The graph measures extracted from rs-fMRI functional connectivity were used as features. Extracted network-based features were fed to the RFE feature selection algorithm to select the most discriminating subset of features. We trained and tested Support Vector Machine (SVM), Random Forest (RF), and Gradient Boosting (GB) using Peking center data from ADHD-200 database to classify ADHD and TD children using discriminative features. In addition to the machine learning approach, the statistical analysis was conducted on graph measures to discover the differences in the brain network of patients with ADHD.

**Results:**

An accuracy of 78.2% was achieved for classifying drug-naive children with ADHD from TD controls employing the optimal features and the GB classifier. We also performed a hub node analysis and found that the number of hubs in TD controls and ADHD children were 8 and 5, respectively, indicating that children with ADHD have disturbance of critical communication regions in their brain network. The findings of this study provide insight into the neurophysiological mechanisms underlying ADHD.

**Conclusion:**

Pattern recognition and graph measures of the brain networks, based on the rs-fMRI data, can efficiently assist in the classification of ADHD children from TD controls.

## Introduction

Attention-Deficit / Hyperactivity Disorder (ADHD) is one of the most common childhood psychiatric disorders. According to the fifth edition of the Diagnostic and Statistical Manual of Mental Disorders (DSM-5) diagnostic criteria, ADHD is defined as inattention and/or hyperactivity/impulsivity. Children with ADHD may have problems concentrating on a specific task or sitting quietly without fidgeting for long periods. The most prominent ADHD phenotypes are ones with predominantly inattentive presentation (ADHD-I) and those that combine inattention and hyperactivity/impulsivity symptoms (ADHD-C) (Epstein and Loren, 2013). ADHD typically begins in childhood and continues into adolescence and adulthood, affecting 3–5% of school-age children. Children with ADHD show behavioral problems and lack of concentration, which make them highly vulnerable to poor academic and social performance (Leitner, 2014).

Today, the combination of modern network science and neuroimaging techniques has made it possible to study the functional network of the human brain (Hakimdavoodi and Amirmazlaghani, 2020). The resting-state fMRI (rs-fMRI), a non-invasive and sensitive method to detect changes in functional brain networks, has attracted considerable attention in the study of brain networks. Since cognitive and behavioral processes depend on large-scale network communications, resting-state functional connectivity analysis may elucidate fundamental aspects of ADHD pathophysiology (Sanchez-Alonso et al., 2021).

In recent years, graph approaches have also gained much popularity in studying the structural and functional connectivity of the brain at various scales for mapping human brain networks. Graph theoretical approaches provide a mathematical framework in which graphs comprise a set of nodes and edges. In the brain, nodes may correspond to predefined brain regions, whereas edges represent functional pairwise correlations among those nodes. While previous studies demonstrated the ability of rs-fMRI in identifying patients with ADHD from healthy controls, the utility of graph measures obtained from rs-fMRI data in the classification of ADHD from TD controls, has not been completely explored (dos Santos Siqueira et al., 2014; Miao et al., 2019; Shao et al., 2020).

Despite the remarkable success of machine learning approaches in rs-fMRI for disease diagnosis, automatic classification of patients with ADHD from typical development subjects using graph measures obtained from rs-fMRI data as interpretable biomarkers has received little attention in ADHD neuroimaging studies (Dai et al., 2012; Miao and Zhang, 2017). Furthermore, these studies have used a limited number of discriminative features such as correlation coefficients among particular brain regions, the amplitude of low-frequency fluctuations (ALFF), and regional homogeneity (ReHo) (Colby et al., 2012; An et al., 2013; Miao et al., 2019) and have not obtained a good accuracy since the employed features only consider specific local characteristics and neglect the dynamics aspects of the whole brain. The primary contribution of the present study is to propose an automatic and precise approach for the classification of patients with ADHD from typical development controls. We conducted a functional connectivity analysis and subsequently obtained the graph measures for children in both healthy and ADHD groups. Then we statistically compared these measures to detect functional aberrations of brain regions during ADHD. The classification method used in this study consisted of four steps of preprocessing, feature extraction, feature selection, and classification. The extracted features are a different set of integration, segregation, and local brain network measures.

Since neuroimaging evidence suggests that the characteristics of the small-world network are affected by neurodevelopment disorders, the optimal balance between global integration and local segregation may be disturbed during ADHD (Wang et al., 2009; Xia et al., 2014). Thus, we added small-world network parameters (i.e., characteristic path length) to our extracted brain network measures. We have used three different machine learning algorithms to evaluate which classifier had the highest performance. ADHD medications such as methylphenidate and amphetamines, which are first-line medical treatments for ADHD, may affect the brain functional connections of children (Pereira-Sanchez et al., 2021). Therefore, we studied drug-naive children in present study. We hypothesized that the proposed approach in the current study could accurately classify children with ADHD from TD controls. In addition, the graph-dependent features selected in the feature selection step not only be used in the machine learning approach but also appear as biomarkers in statistical analysis to provide information about ADHD-induced functional networks. Considering the importance of reproducibility in the results of neuroimaging studies using MRI, we organized the current research according to the Organization for Human Brain Mapping (OHBM) recommendations (Nichols et al., 2017).

## Materials and methods

### Participants

The present study used resting-state fMRI data obtained from the publicly available ADHD-200 Consortium.^1^ The ADHD database consists of eight different centers, making the data diverse and complex. Peking University was selected for this study because of the large sample size. It includes 143 TD subjects and 102 children with ADHD. A total of 112 right-handed subjects between the ages of 8 to 17, including 56 patients with ADHD, were selected from the Peking center according to the DSMIV-TR criteria and 56 typical development (TD) controls after applying the inclusion criteria.

Psychiatric diagnoses were verified at Peking University through psychiatric interviews with experienced child psychiatrists using the Schedule of Affective Disorders and Schizophrenia for Children–Present and Lifetime Version (K-SADS-PL) administered to parents and children, and the ADHD Rating Scale-IV. Intelligence was evaluated with the Wechsler Intelligence Scale for Chinese Children-Revised (WISCC-R). The following exclusion criteria were applied to all participants: (1) a history of conduct disorder, oppositional defiant disorder, Tourette’s disorder, and any neurologic disorder that could impact the functional connectivity patterns of the brain; (2) a score of less than 80 for the full-scale WISCC-R; (3) a mean frame-wise displacement (FD) more than 0.3 mm; and (4) missing even one of the clinical phenotype listed in Table 1. All research protocols from institutes contributing to the ADHD-200 Consortium received local approval from their respective IRB. All the data distributed *via* the International Neuroimaging Data-sharing Initiative (INDI) are fully anonymized by HIPAA Privacy Rules.

**TABLE 1:** Demographic and clinical characteristics of ADHD and TD control groups.

Clinical phenotype	TD (*n* = 56)		ADHD (*n* = 56)		ADHD vs. TD
			
	Mean	SD	Mean	SD	*T*-values
Age (years)	11.51	1.77	11.86	2.21	0.920
Full IQ	118.2	13.46	103.55	12.63	−5.941***
Performance IQ	110.93	15.03	98.43	11.86	−4.885***
Verbal IQ	120.9	13.26	107.48	15.25	−4.991***
FD	0.1434	0.058	0.152	0.046	0.853
Gender (Male/Female)	**44/12**		**49/7**		χ^2^ **1.585**

***p < 0.001.

TD, typical development controls; ADHD, attention-deficit/hyperactivity disorder; FD, frame-wise displacement; IQ, intelligence quotient.

### Image acquisition

High-resolution whole-brain T1-weighted 3D MPRAGE data were acquired for each participant on a SIEMENS TRIO 3-Tesla MRI scanner with the following imaging parameters: repetition time (TR) = 1700 ms, echo time (TE) = 3.92 ms, field of view (FOV) = 256 mm × 256 mm, 176 slices, thickness/gap = 1.0, and voxel resolution = 1.0 mm × 1.0 mm × 1.0 mm; flip angle = 12°. The rs-fMR imaging was obtained axially using a blood oxygenation level-dependent contrast sensitive gradient echo-planar imaging (TR = 2,000 ms, TE = 30 ms, flip angle = 90°, FOV = 220 mm × 220 mm, 236 total time points (about 8 min), 220 mm × 220 mm (FOV), 64 × 64 (resolution), 30 slices, 4.5/0 mm thickness/gap. During the acquisition of functional data, participants were asked to relax with their eyes close and not to concentrate on anything in particular.

### Data pre-processing

Resting-state fMRI data preprocessing was carried out using the GRETNA toolbox and SPM12 (Wang et al., 2015). The first 10 functional volumes were removed to allow T1 equilibration effects. The remaining volumes were corrected for within-volume timing acquisition differences among slices (Sinc interpolation) and inter-volume head-motion effects. Functional MRI volumes (time-series) realigned using a six-parameter rigid-body spatial transformation to compensate for head-motion effects. Following the head-motion correction, the individual T1-weighted MPRAGE images were co-registered to the mean functional image employing a linear transformation. The transformed structural images were then segmented into gray matter (GM), white matter (WM), and cerebrospinal fluid (CSF) using a unified segmentation algorithm. The head-motion corrected images were then spatially normalized into standard Montreal Neurological Institute (MNI) space through segmentation and resampled to 3-mm isotropic voxels. The normalized images were subsequently subjected to removal of linear trends and temporal band-pass filtering (0.01–0.1 Hz), which were employed to decrease the effects of low-frequency fluctuation and high-frequency physiological noises, respectively. To further reduce the head-motion effects, a scrubbing method was conducted on each subject’s fMRI time series to discover the mean frame-wise displacement that was higher than 0.5 mm (Power et al., 2012). In this method, one volume before and two volumes after the ineligible volume were linearly interpolated. Finally, some nuisance signals, including the WM signal, CSF signal, global signal, and 24 head-motion parameters were extracted and regressed out from each voxel’s time series, and then the data was smoothed using a 2*(voxel-size) mm full width half maximum (FWHM) Gaussian filter (Kelly et al., 2008).

### Network construction

**FIGURE 1:**
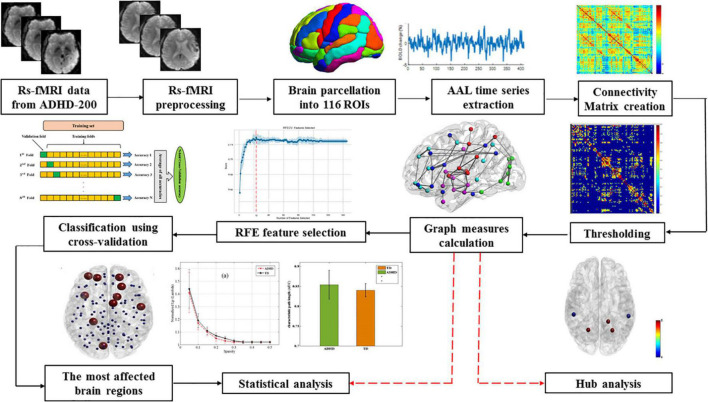
The overall procedure of this study.

The overall procedure of the current study can be seen in Figure 1. Following the preprocessing of rs-fMRI data, the Automated Anatomical Labeling (AAL) atlas was used to parcel each participant’s brain into 116 anatomical regions of interest (ROIs). Each region’s average time series was then calculated by averaging the time series of all voxels within the ROIs.

To create a 116 × 116 symmetric correlation matrix for each subject, Pearson correlation was calculated between each pair of regional time series. Finally, to explore the properties of brain functional connectivity (functional networks), all the correlation matrices were thresholded into a binary graph with a fixed density level for further graph theory-based analyses, where nodes stand for brain regions and edges represent undirected connections between nodes. Fixed network density is the ratio of the actual number of edges divided by the maximum feasible number of network edges. Thus, this thresholding approach makes it possible to investigate the relative network organization by imposing on each network the same number of edges or wiring costs for compensatory compatibility. Since there is no specific method for selecting a unique threshold, we utilized a wide range of cost values (i.e., 10–50%) in steps of 1%.

### Graph measures and network analysis

The whole-brain functional network (functional connectivity) analysis was conducted using the GRETNA toolbox (Wang et al., 2015). Graph theory was used to calculate the global and local graph measures to classify healthy individuals from ADHD patients. The local graph measures used in this study included betweenness centrality, clustering coefficient, (shortest) path length, local efficiency, and degree centrality. Global graph measures were assortativity, global efficiency, small-world, synchronization, and hierarchy. Furthermore, the small-world parameters such as the clustering coefficient (Cp) and path length (Lp), were calculated in terms of Watts and Strogatz (1998). Briefly, the characteristic path length of a network is defined as the average number of edges in the shortest paths connecting any two nodes in the network. The path length is employed to calculate how well a network is connected, and a low value represents an average short distance between any two nodes. The clustering coefficient is defined as the number of actual edges linking the neighbors of a node divided by the maximum number of edges possible between neighboring nodes. The clustering coefficient is used to calculate the number of local clusters in the network. To discover the characteristics of the small-world, the Cp and Lp values of the functional brain network must be compared with those values of random networks [C (rand) and L (rand) respectively]. Therefore, One hundred random networks that maintain an equal number of nodes, edges, and degree distributions were generated as real networks using the Markov-chain algorithm (Maslov and Sneppen, 2002; Milo et al., 2002).

To further explain the characteristics of the small-world network, some parameters such as gamma and sigma were defined. Networks with small-world properties have a relatively high gamma, which is defined as follows:


(1) $$\gamma =\frac{C\,p}{C\,\left(r\,a\,n\,d\right)}>1$$

Also, these networks have almost the same Lp compared to random networks. Therefore, the sigma in the networks with small-world properties becomes more than one.


(2) $$\lambda =\frac{L\,p}{L\,\left(r\,a\,n\,d\right)}\approx 1$$

(3) $$\delta =\frac{\gamma }{\lambda }>1$$

Graph theoretical approaches can provide valuable insights into network efficiency in terms of how information is transferred across a network. Local efficiency indicates the information flow transmission in a network at the local level. It provides insight into the segregated ability of the brain network (Rubinov and Sporns, 2010). Consequently, global efficiency measures the capability of the brain network to globally parallel information transmission. High local and global efficiencies represent more efficient information propagation across a local and global network, respectively (Ma et al., 2018). In a network, nodes that maintain a different set of inter-modular connections and facilitate integration between modules are known as hubs. In the current study, the hub nodes were calculated for both ADHD and TD children based on degree and betweenness centrality. In two groups, the degree and betweenness centrality for each node were calculated, and then nodes that were larger than average by more than two standard deviations were determined as hubs (Rubinov and Sporns, 2010).

### Feature selection and classification

Using a feature selection algorithm is the necessary part of a machine learning technique, which promotes data interpretation, and subsequently improves the performance of a classification system. Recursive Feature Elimination (RFE) is a robust algorithm for feature selection presenting a precise way to determine the meaningful features before using them as input for a machine learning algorithm. RFE uses all features to construct a classification model. Then, it ranks the cooperation features in the classification model into a ranked feature list. Finally, RFE eliminates the irrelevant features that have a senseless collaboration with the classification model (Bahl et al., 2019).

The classification of ADHD children from TD controls was conducted using three different classifiers, including support vector machine (SVM), gradient boosting (GB), and random forest (RF). Then, these supervised machine learning algorithms were trained using a set of input data to yield the desired output. Since the obtained classification accuracy by SVM with linear kernels was similar to that of non-linear kernels and considering that solving the optimization problem for SVM with the linear kernel is much faster than the non-linear kernel, in this study SVM with a linear kernel was used. The number of available data in imaging research is usually low due to the high cost of data acquisition. Therefore, different cross-validation methods have been proposed to overcome the loss of generalization resulting from the small training and testing sample size. Since age, sex, race, handedness, and imaging parameters can be potential confounding variables in examining subjects’ brain network topology, we selected training and testing data only from the Peking center to not only classify drug-naïve ADHD children from TD controls but also more accurately investigate the functional brain changes in ADHD children.

**FIGURE 2:**
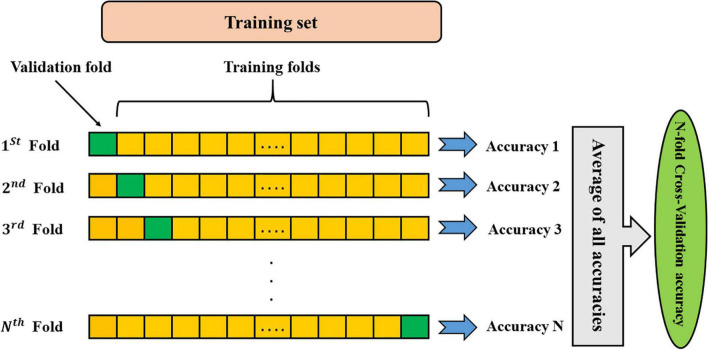
Schematic representation of K-fold cross-validation method. Data colored as yellow are the training folds, and the green fold is the one that leaved out for the validation.

The k-fold cross-validation strategy presents a trade-off between bias and error variance. Therefore, selecting optimal values for “k” ensures that the selected method provides a suitable estimate of the model performance. If the value of k is small, cross-validation can lead to a lot of bias for error. The k-fold cross-validation with a high value of k can lead to a lot of error variance and is computationally intensive. In machine learning scenarios, the value of k is usually used between 5 and 10. In the present study, the best accuracy was achieved using k-fold cross-validation of 9. The procedure is iterated until all subjects are considered once as the test sample. Eventually, the results of all repetitions are averaged to provide the ultimate classification accuracy (Figure 2).

### Circular analysis

Using the data of one center as training and test data may reduce the generalizability of a classification model and cause inflation of accuracy in the test data due to an error of including all data when selecting features for the classification model. For the classifier to be insured free of circular analysis, we used an iterative cross-validation procedure where the feature selection (RFE) step was performed using the GF model only on the training set of every iteration.

### Statistical analysis

Statistical analyses were performed in SPSS 16.0. For each continuous variable, normal distribution was assessed by the Shapiro-Wilk test. Statistical group differences between the ADHD children and typical development children in age, sex, and all type of IQs were determined using *t*-tests. Group differences in small-world properties were assessed applying ANCOVA, with age, sex, FD, and IQs as covariates. Throughout, multiple comparisons were controlled using the false discovery rate (*p* < 0.05).

## Results

### Demographic and clinical characteristics

As mentioned above, Table 1 summarizes the characteristics of ADHD and TD children. Compared with the control group, the ADHD children showed a higher mean age (ADHD: mean = 11.86; TD: mean = 11.51, *T* = 0.920, *p* > 0.05), a lower mean IQ (ADHD: mean = 103.21; TD: mean = 116.95, *T* = –6.35, *p* < 0.001), more males (χ^2^ = 1.585, *p* > 0.05) and more head-motion (ADHD: mean = 0.152; TD: mean = 0.1434, *T* = 0.853, *p* > 0.05). There were no significant differences in age and FD between the two groups, as detailed in Table 1. In the subsequent analysis, age, gender, FD, verbal, performance, and full-scale IQs were used as covariates.

### Small-world topology of brain functional networks

**FIGURE 3:**
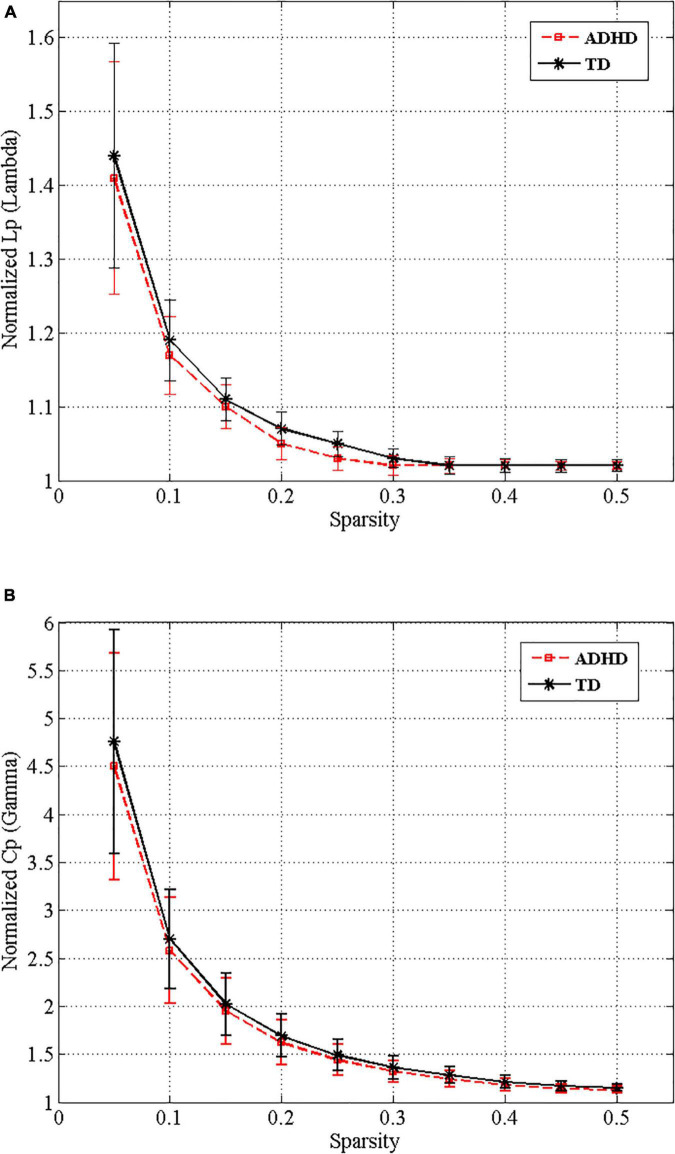
Small-world properties of the functional whole-brain networks of drug-naive ADHD children and typical development (TD) controls over the different sparsity range. Normalized shortest path length **(A)** and normalized clustering coefficient **(B)** of ADHD (red dashed line) and TD (black line) groups.

**FIGURE 4:**
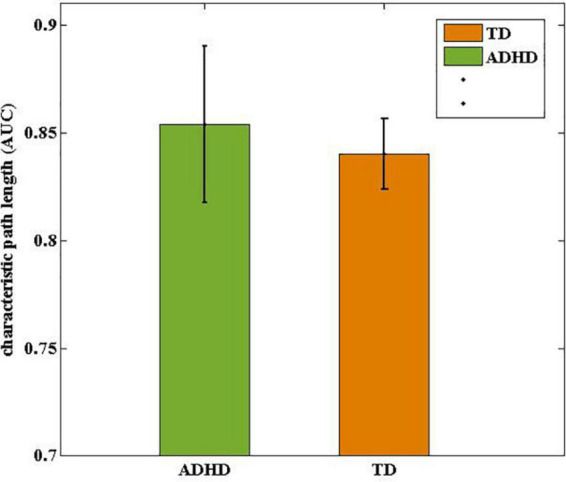
Comparisons of drug-naive ADHD children and TD controls in terms of characteristic path length (Lp). The characteristic path length in ADHD group was statistically longer than in the TD group.

Over the entire range of sparsity values, the normalized path length was approximately 1 [equation (2) and (Figure 3A)], and the normalized clustering coefficient was greater than 1 [equation (1) and (Figure 3B)]. These findings demonstrated the economic small-world topology of the brain functional network in both groups [equation (3)]. After controlling for age, gender, FD, and all types of IQs using ANCOVA, compared with the TD group, the ADHD children exhibited an increase in the characteristic path length (9.8 × 10^–3^, FDR corrected) (Figure 4). However, there were no significant differences in the clustering coefficient (Cp), the normalized clustering coefficient (γ), normalized path length (λ), or the small-world (σ).

### Discrimination of attention-deficit/hyperactivity disorder children and typical development controls

Different classifiers, such as RF, SVM, and GB have been used in many machine learning based neuroimaging studies. We investigated the performance of these classifiers for discrimination of ADHD children from TD controls. The best performance was achieved using the gradient boosting classifier. The performance of the classifiers was compared by employing a nine-fold cross-validation strategy in Table 2. The gradient boosting classifier outperformed the SVM and RF classifiers in all the performance metrics and achieved a high accuracy of 78.2%. Table 3 represents the performance of the classifiers after assessing the contribution of circular analysis in our data.

**TABLE 2:** Classification performance of three different classifiers using a subset of optimal features, extracted from rs-fMRI using AAL atlas.

Classifier	Accuracy (%)	Sensitivity (%)	Specificity (%)	PPV (%)	NPV (%)
GB	78.2	75	80	66.6	85.7
RF	69.5	87.5	60	46.6	90
SVM	56.5	75	46.6	42.8	77.7

PPV, positive predictive value; NPV, negative predictive value; GB, gradient boosting; RF, random forest; SVM, support vector machine.

**TABLE 3:** Classification performance of three different classifiers using a subset of optimal features, extracted from rs-fMRI using AAL atlas after assessing the contribution of circular analysis.

Classifier	Accuracy (%)	Sensitivity (%)	Specificity (%)	PPV (%)	NPV (%)
GB	74.2	69.8	78.6	79.1	71.7
RF	69.2	68	70.4	72.9	68
SVM	51.8	62.4	41.3	50.5	57.3

**FIGURE 5:**
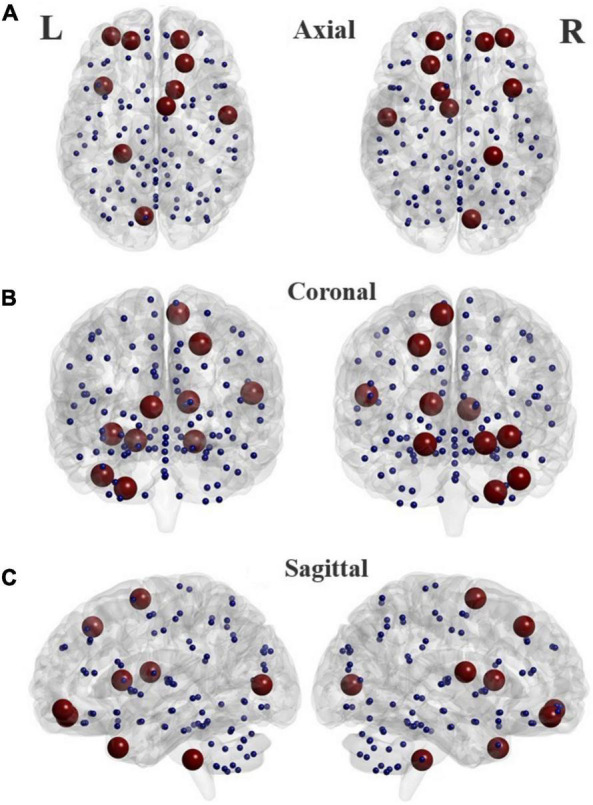
Locations of the brain regions in the AAL atlas (listed in Table 4) that present highest discrimination ability between TD and ADHD groups in different planes. Axial **(A)**, Coronal **(B)**, and Sagittal **(C)**.

The top twelve features corresponding to the best classifier (GB classifier), are listed in Table 4. Of those features, two global, and three nodal graph measures were statistically different in the two groups. Three nodal rs-fMRI right graph measures, i.e., betweenness centrality in the middle frontal gyrus (orbital part), cerebellum, and nodal efficiency in the left rolandic operculum of ADHD children were significantly higher than that of TD controls (*p* < 0.032). The global efficiency (a global graph measure) of the ADHD group was significantly lower, whereas local efficiency was significantly higher than that of TD controls (*p* < 5.7 × 10^–5^). Locations of the AAL brain regions that show highest discrimination ability corresponding to the optimal subset of graph measures are shown in Figure 5.

**TABLE 4:** Mean, standard deviation, and statistical comparison of top 12 graph features selected by RFE in children with ADHD and TD groups.

Graph features	Type of graph features	Brain regions	MNI coordinates			Resting-state network	ADHD	TD	*p*-value
							
			X	Y	Z				
Betweenness centrality	Nodal	Frontal Mid Orb L	−31	50	−10	FPN	16.41 ± 11.53	9.04 ± 4.2	**2.79 × 10** ^–^ **^3^**
	Nodal	Cerebellum_10 L	−22	−34	−42	Cerebellum	11.65 ± 9.08	5.74 ± 2.07	**1.82 × 10^–2^**
Global efficiency	Global	–	–	–	–	–	0.246 ± 0.011	0.259 ± 0.011	**1.89 × 10^–8^**
Local efficiency	Global	–	–	–	–	–	0.350 ± 0.009	0.342 ± 0.010	**5.66 × 10^–5^**
Nodal efficiency	Nodal	Rolandic Oper R	53	−6	15	Auditory/cingulo-opercular	0.255 ± 0.023	0.244 ± 0.027	**3.17 × 10^–2^**
	Nodal	Supp motor area R	9	0	62	Ventral attention	0.273 ± 0.033	0.266 ± 0.035	2.5 × 10^–1^
Nodal local efficiency	Nodal	Frontal Sup Orb L	−17	47	−13	FPN	0.339 ± 0.029	0.349 ± 0.030	1.14 × 10^–1^
	Nodal	Frontal Sup Orb R	18	48	−14	FPN	0.336 ± 0.042	0.347 ± 0.043	1.82 × 10^–1^
	Nodal	Calcarine L	−7	−79	6	Visual	0.374 ± 0.027	0.368 ± 0.025	2.64 × 10^–1^
	Nodal	Temporal Pole Mid L	−36	15	−34	DMN	0.359 ± 0.053	0.369 ± 0.036	2.41 × 10^–1^
Nodal path length	Nodal	Frontal Sup R	22	31	44	FPN	0.741 ± 0.073	0.789 ± 0.272	1.97 × 10^–1^
	Nodal	Caudate R	15	12	9	subcortical	7.89 ± 4.8	7.64 ± 5.13	8.67 × 10^–1^

MNI, Montreal neurological institute.

Frontal Mid Orb: Orbital part of middle frontal gyrus; Rolandic Oper: Rolandic operculum; Supp Motor Area: Supplementary motor area; Frontal Sup Orb: Orbital part of superior frontal gyrus; Temporal Pole Mid: Temporal pole middle temporal gyrus; Frontal Sup: dorsolateral superior frontal gyrus; Caudate: Caudate nucleus; R: Right hemisphere; L: Left hemisphere. The bold values indicate a statistically significant difference with a *p*-value < 0.05.

### Identifying hub nodes

**FIGURE 6:**
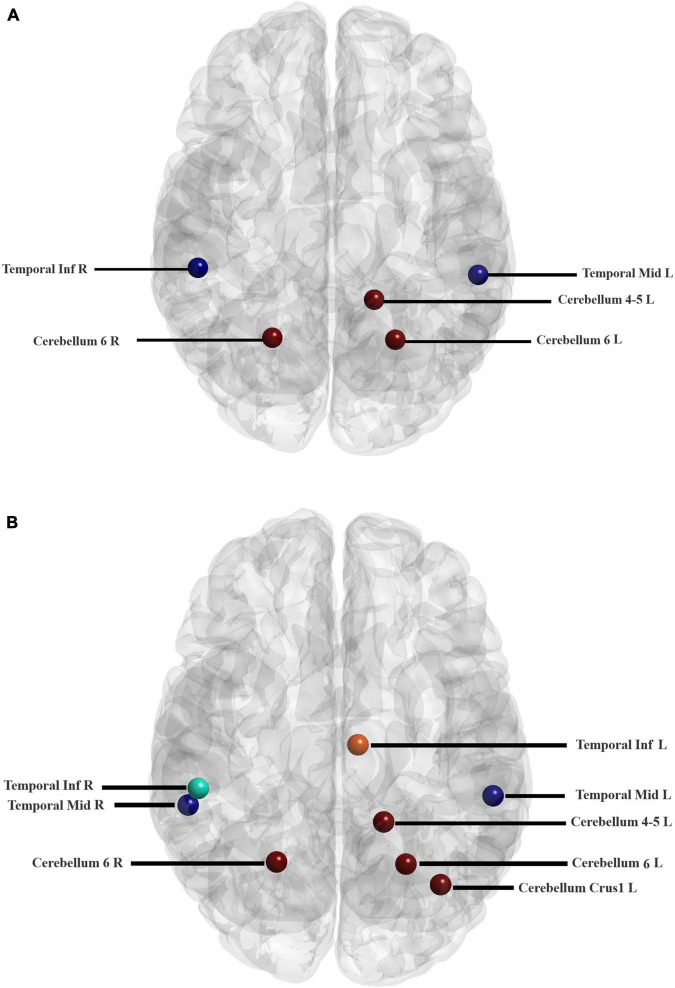
Locations of the hub regions of two groups in the AAL atlas (listed in Table 5). Hub regions of ADHD children **(A)** and TD group **(B)**.

The hub nodes were determined in two groups of children, i.e., ADHD, and TD. Nodes with a betweenness centrality of two standard deviations higher than the average of the betweenness centrality of all nodes were identified as hub regions. As listed in Table 5, some hub nodes are identical between two groups, namely ROIs 85, 90, 97, 99, and 100, which correspond to the left middle temporal gyrus, right Inferior temporal gyrus, left cerebellum 4–5, left cerebellum 6, and right cerebellum 6, respectively. In addition, the corresponding AAL area and resting-state network of each hub region are given in the table. More notably, several hub nodes are present only in the TD group, missing in the ADHD group. These hub nodes are within the left median cingulate and paracingulate gyri, left cerebellum crust 1, and right middle temporal gyrus. It is noteworthy that all hub nodes in the ADHD group were also observed in the TD group (Figure 6).

**TABLE 5:** The brain regions and resting-state networks related to hub nodes calculated based on Betweenness centrality in ADHD and TD groups.

	ROI no	Corresponding brain region in AAL atlas	Resting-state network
Common hubs in TD and ADHD	85	Left middle temporal gyrus	DMN
	90	Right Inferior temporal gyrus	FPN
	97	Left cerebellum 4_5	Cerebellum
	99	Left cerebellum 6	Cerebellum
	100	Right cerebellum 6	Cerebellum
Hubs only in TD	33	Left median cingulate and paracingulate gyri	Salience/cingulo-opercular
	86	Right middle temporal gyrus	DMN
	91	Superior lobe of left cerebellum	Cerebellum

## Discussion

The small-world characteristics of functional networks of the human brain have been confirmed by different imaging modalities such as EEG, MEG, and fMRI (Bullmore and Sporns, 2012; Stam, 2014). The current study used binary graphs to investigate resting-state functional MRI data and exhibited large-scale functional alterations in the organization of drug-naive ADHD children’s brain networks. Consistent with findings from previous studies on functional brain connectivity in children with ADHD, we found an economic small-world organization across a range of sparsity in both the ADHD and TD children groups, which proposes that small-world brain networks are resistant to developmental disorder (Figure 3; Cocchi et al., 2012; Chen et al., 2019). According to previous studies, although the brain functional networks of children in both study groups have the economic small-world characteristics, the reports about the topological alterations in the small-world properties are not consistent (Wang et al., 2009; Xia et al., 2014; Tao et al., 2017).

We found that the characteristic path length in ADHD group was longer than in the TD group (Figure 4), which is in line with Chen et al. study, while Cocchi et al. reported that there was no significant difference in path length in ADHD children compared to healthy individuals (Cocchi et al., 2012; Chen et al., 2019). It is noteworthy that due to the heterogeneity in participant characteristics, experimental methods, analytical flexibility, and also the complex pathophysiology of ADHD, the results of the ADHD studies do not have significant spatial convergence (Cortese et al., 2021). Global efficiency and characteristic path length are mainly affected by shorter and longer paths, respectively. Recent studies suggest that reduced functional connectivity in children with ADHD is primarily due to reduced long-range connections of the brain (Tao et al., 2017). Therefore, our findings of reduced global efficiency and increased characteristic path length in drug-naive ADHD children may indicate an increase in long-range connections, reflecting the brain’s compensatory adaptations response to ADHD pathophysiology problems.

Also, we used resting-state fMRI and combined the graph-theoretical approach with the machine learning methods to classify drug-naive ADHD children from typical development (TD) controls. We used three different classifiers (SVM, GB, and RF) with a small number of graph measures selected by a recursive feature elimination algorithm and achieved high accuracy in classifying ADHD children from TD controls. Our results revealed that the gradient boosting classifier outperformed the two other classifiers in terms of accuracy, sensitivity, and positive predictive value (Table 2). The high accuracy values in neuroimaging classification studies in ADHD have raised concerns about the methodological robustness. Recent report by Pulini et al. (2019) indicate using all subjects’ data rather than the training subset only in the feature selection step may cause a circularity error which can inflate the accuracy of the test data. Therefore, we assessed the contribution of circular analysis to classification accuracy to investigate how much the obtained accuracy was affected by methodological factors in our study. The results presented in Table 3 confirm the robustness of our methodology because after addressing the circularity error, the accuracy of GB and SVM classifiers decreased by only about 4%. It is noteworthy that the accuracy of the RF classifier almost did not change after the circular analysis.

The GB is an ensemble of the decision tree model. First, a simple model is created on the data then the model error is evaluated. If the error was not reduced enough, another model is added to the old model to decrease the error. This operation continues until the desired error is obtained. Therefore, the gradient boosting algorithm can learn complex patterns (Hastie et al., 2009). In RF classifier, the data (features) are modeled using a set of trees. Each tree is unrelated to the errors of other trees and models only its training data (Hastie et al., 2009). Therefore, RF may not be able to perform better than the GB classifier. Regarding the weakness of SVM results compared to the other two models, it can be said that the feature space may be divided into hyper-rectangles between two classes. Tree-based classifiers such as RF and GB model the feature space using thresholding on individual features, while the SVM works based on linear and non-linear functions on a feature set.

Table 2 shows that the GB classifier provided a superior accuracy compared to previous studies in ADHD classification, indicating that graph measures derived from rs-fMRI could be as promising biomarkers in the diagnosis of children with ADHD (Colby et al., 2012; Shao et al., 2020). Dos Santos Siqueira et al. used graph theory and rs-fMRI data to classify ADHD from TD controls. In their study, only graph centrality features were used as input to SVM, and the maximum classification score was 55% for the center of Peking University (PU), which was slightly lower than the accuracy obtained by SVM in our study (56.5%). Higher accuracy in the present study can be related to the number of graph measures examined and applying the feature selection algorithm, which was not used in the study of dos Santos Siqueira et al. (2014). In another study Shao et al. used functional connectivity measures to classify ADHD from TD controls using RF classifier. The accuracy of RF classifier for PU center was obtained about 67.2%, which was lower than the accuracy obtained by RF classifier in our study (69.5%) (Shao et al., 2020).

The top 12 graph features with the most discrimination capability in Table 4 are primarily associated with areas in the frontoparietal network (FPN). FPN is one of the main brain resting-state networks involved in ADHD pathophysiology and has a prominent role in the flexible utilization of cognitive control and considerable top-down cognitive processes. The frontoparietal network is repeated four times in Table 4, corresponding to three graph measures, i.e., betweenness centrality, nodal local efficiency, and nodal path length. This result is consistent with several studies that have reported abnormal functional connectivity within and between FPN, default mode network (DMN), and attention networks (dorsal, ventral, salience) in ADHD (Castellanos and Aoki, 2016; Gao et al., 2019; Wang et al., 2019; Cortese et al., 2021). Also, as it can be seen, features associated with the left cerebellum (which contributes to motor function and cognitive processing), right rolandic operculum (which involves the language processing system, motor, sensory, and cognitive processing), right supplementary motor area (which involves in executive functions), calcarine fissure, left middle temporal gyrus of the temporal pole, and right caudate nucleus (which involve in planning the movement execution, learning, memory, reward, motivation, and emotion) are among the top discriminative features. Consistent with our findings, brain functional alterations in these regions particularly in the middle temporal gyrus in patients with ADHD have been reported in previous rs-fMRI studies (Wang et al., 2009; dos Santos Siqueira et al., 2014; Lui et al., 2016; Ardila et al., 2018; Marcos-Vidal et al., 2018; Cortese et al., 2021).

Studies on various brain disorders have revealed that brain lesions are mostly located in the hub areas, hence investigating the hub areas may be a suitable approach for understanding brain disorders (Menon, 2013; Crossley et al., 2014).

As listed in Table 5, three hub regions, namely left median cingulate, paracingulate gyri (frontal lobe), right middle temporal gyrus (temporal lobe), and the superior lobe of the left cerebellum (cerebellum), were not present in drug-naive ADHD children, whereas TD controls had these hubs. The importance of the role of the cingulate cortex has been shown in the executive function. Executive functions are a collection of cognitive processes that comprise primary cognitive processes such as working memory, attentional control, cognitive flexibility, inhibitory control, and cognitive inhibition. It has been shown that cognitive control is impaired in attention deficit hyperactivity disorder (Friedman and Robbins, 2022). Thus, lack of hub node in left median cingulate, and paracingulate gyri may relate to impairment of executive functions. The lack of a hub node in the right middle temporal gyrus in patients with ADHD may relate to the impairment of guiding the attention control and action in these patients (Shaw et al., 2007). The lack of a hub node in the superior lobe of the left cerebellum in ADHD children may be associated with the key role of the cerebellum in various functions. Since the cerebellum involves in many functions such as motor control, learning, attention shifting, visual-spatial processing, and working memory, it could have a crucial role in the deficient attentional and cognitive control mechanisms underlying ADHD (Brissenden et al., 2018; Schmahmann, 2019; Clark et al., 2021).

## Study limitations and conclusion

Despite all the strengths of this work, there are several limitations to this study should be further addressed. First, we used the structural atlas AAL 116, which is common in functional studies. Since there is no one-to-one correspondence in the definition of functional and structural brain areas, using a functional atlas such as Power 264 atlas can be a more suitable option. Second, we used the binary graph for brain network construction. However, the use of the weighted graph and its unique features may be effective in distinguishing between ADHD and TD controls, as well as providing information on the physiological mechanism of ADHD. Third, we only used the rs-fMRI data in our study. Using multi-modal imaging (MRI, fMRI, and DWI) and non-imaging (e.g., IQ, ADHD severity) data may provide additional information that can better characterize the discrepancies between ADHD children and TD controls. Fourth, since graph theory measures hold potential of clinical significance, they deserve further study in neuroimaging research in the areas of diagnosis, prognosis, and psychopharmacology and therapeutics of ADHD.

We combined the binary graph features obtained from rs-fMRI data with machine learning methods to classify drug-naïve ADHD children from the typical development (TD) controls. Our results showed that in ADHD children, regions located in the frontal lobe (frontoparietal network) undergo changes more than other brain regions. We determined optimal graph features with high discrimination ability using a recursive feature elimination algorithm to provide insights into ADHD pathophysiology. The classifiers were trained and cross-validated, then their performances were evaluated. We found that the best classifier for classifying ADHD children from TD controls obtained an accuracy of 78.2%. We also performed a hub node analysis and found that the number of hubs in TD controls and ADHD children were 8 and 5, respectively, indicating that children with ADHD have the disturbance of critical communication regions in their brain network. These graph features facilitated machine learning algorithms to accurately classify ADHD from TD controls, indicating that graph features should be given more attention as potential biomarkers in neuroscience studies. This study brings an original methodological approach to advance the search for reliable and accurate biomarkers for the diagnosis of ADHD. However, the findings are still far from providing clinical advantages, and there is still uncertainty about the reliability of potential neuroimaging biomarkers for ADHD diagnosis. Therefore, further methodological, neuroscience and translational clinical neuroscience research is needed.

## Data availability statement

The original contributions presented in the study are included in the article/supplementary material, further inquiries can be directed to the corresponding author.

## Ethics statement

This study involving human participants were reviewed and approved by the Research Ethics Committee of School of Medicine Mashhad University of Medical Sciences. Written informed consent from the participants’ legal guardian/next of kin was not required to participate in this study in accordance with the national legislation and the institutional requirements.

## Author contributions

MR and PH conceived the original idea. MR and SN carried out the experiment. HZ and HH supervised the project. SN wrote the manuscript with support from MR. All authors contributed to the article and approved the submitted version.

## Footnotes

1
http://fcon_1000.projects.nitrc.org/indi/adhd200/

## References

[B1] An L. Cao Q.-J. Sui M.-Q. Sun L. Zou Q.-H. Zang Y.-F. (2013). Local synchronization and amplitude of the fluctuation of spontaneous brain activity in attention-deficit/hyperactivity disorder: A resting-state fMRI study. *Neurosci. Bull.* 29 603–613. 10.1007/s12264-013-1353-8 23861089 PMC5561960

[B2] Ardila A. Bernal B. Rosselli M. (2018). Executive functions brain system: An activation likelihood estimation meta-analytic study. *Arch. Clin. Neuropsychol.* 33 379–405. 10.1093/arclin/acx066 28961762

[B3] Bahl A. Hellack B. Balas M. Dinischiotu A. Wiemann M. Brinkmann J. (2019). Recursive feature elimination in random forest classification supports nanomaterial grouping. *NanoImpact* 15:100179. 10.1016/j.impact.2019.100179

[B4] Brissenden J. A. Tobyne S. M. Osher D. E. Levin E. J. Halko M. A. Somers D. C. (2018). Topographic cortico-cerebellar networks revealed by visual attention and working memory. *Curr. Biol.* 28 3364–3372.e5. 10.1016/j.cub.2018.08.059 30344119 PMC6257946

[B5] Bullmore E. Sporns O. (2012). The economy of brain network organization. *Nat. Rev. Neurosci.* 13 336–349. 10.1038/nrn3214 22498897

[B6] Castellanos F. X. Aoki Y. (2016). Intrinsic functional connectivity in attention-deficit/hyperactivity disorder: A science in development. *Biol. Psychiatry Cogn. Neurosci. Neuroimaging* 1 253–261. 10.1016/j.bpsc.2016.03.004 27713929 PMC5047296

[B7] Chen Y. Huang X. Wu M. Li K. Hu X. Jiang P. (2019). Disrupted brain functional networks in drug-naïve children with attention deficit hyperactivity disorder assessed using graph theory analysis. *Hum. Brain Mapp.* 40 4877–4887. 10.1002/hbm.24743 31361385 PMC6865592

[B8] Clark S. V. Semmel E. S. Aleksonis H. A. Steinberg S. N. King T. Z. (2021). Cerebellar-subcortical-cortical systems as modulators of cognitive functions. *Neuropsychol. Rev.* 31 422–446. 10.1007/s11065-020-09465-1 33515170

[B9] Cocchi L. I Bramati E. Zalesky A. Furukawa E. Fontenelle L. F. Moll J. (2012). Altered functional brain connectivity in a non-clinical sample of young adults with attention-deficit/hyperactivity disorder. *J. Neurosci.* 32 17753–17761. 10.1523/JNEUROSCI.3272-12.2012 23223295 PMC6621678

[B10] Colby J. B. Rudie J. D. Brown J. A. Douglas P. K. Cohen M. S. Shehzad Z. (2012). Insights into multimodal imaging classification of ADHD. *Front. Syst. Neurosci.* 6:59. 10.3389/fnsys.2012.00059 22912605 PMC3419970

[B11] Cortese S. Aoki Y. Y. Itahashi T. Castellanos F. X. Eickhoff S. B. (2021). Systematic review and meta-analysis: Resting-state functional magnetic resonance imaging studies of attention-deficit/hyperactivity disorder. *J. Am. Acad. Child Adolesc. Psychiatry* 60 61–75. 10.1016/j.jaac.2020.08.014 32946973

[B12] Crossley N. A. Mechelli A. Scott J. Carletti F. Fox P. T. McGuire P. (2014). The hubs of the human connectome are generally implicated in the anatomy of brain disorders. *Brain* 137 2382–2395. 10.1093/brain/awu132 25057133 PMC4107735

[B13] Dai D. Wang J. Hua J. He H. (2012). Classification of ADHD children through multimodal magnetic resonance imaging. *Front. Syst. Neurosci.* 6:63. 10.3389/fnsys.2012.00063 22969710 PMC3432508

[B14] dos Santos Siqueira A. Biazoli Junior C. E. Comfort W. E. Rohde L. A. Sato J. R. (2014). Abnormal functional resting-state networks in ADHD: Graph theory and pattern recognition analysis of fMRI data. *Biomed Res. Int.* 2014:380531. 10.1155/2014/380531 25309910 PMC4163359

[B15] Epstein J. N. Loren R. E. (2013). Changes in the definition of ADHD in DSM-5: Subtle but important. *Neuropsychiatry* 3:455. 10.2217/npy.13.59 24644516 PMC3955126

[B16] Friedman N. P. Robbins T. W. (2022). The role of prefrontal cortex in cognitive control and executive function. *Neuropsychopharmacology* 47 72–89. 10.1038/s41386-021-01132-0 34408280 PMC8617292

[B17] Gao Y. Shuai D. Bu X. Hu X. Tang S. Zhang L. (2019). Impairments of large-scale functional networks in attention-deficit/hyperactivity disorder: A meta-analysis of resting-state functional connectivity. *Psychol. Med.* 49 2475–2485. 10.1017/S003329171900237X 31500674

[B18] Hakimdavoodi H. Amirmazlaghani M. (2020). Using autoregressive-dynamic conditional correlation model with residual analysis to extract dynamic functional connectivity. *J. Neural Eng.* 17:035008. 10.1088/1741-2552/ab965b 32454472

[B19] Hastie T. Tibshirani R. Friedman J. H. Friedman J. H. (2009). *The Elements Of Statistical Learning: Data Mining, Inference, And Prediction.* Berlin: Springer. 10.1007/978-0-387-84858-7

[B20] Kelly A. C. Uddin L. Q. Biswal B. B. Castellanos F. X. Milham M. P. (2008). Competition between functional brain networks mediates behavioral variability. *Neuroimage* 39 527–537. 10.1016/j.neuroimage.2007.08.008 17919929

[B21] Leitner Y. (2014). The co-occurrence of autism and attention deficit hyperactivity disorder in children–what do we know? *Front. Hum. Neurosci.* 8:268. 10.3389/fnhum.2014.00268 24808851 PMC4010758

[B22] Lui S. Zhou X. J. Sweeney J. A. Gong Q. (2016). Psychoradiology: The frontier of neuroimaging in psychiatry. *Radiology* 281 357–372. 10.1148/radiol.2016152149 27755933 PMC5084981

[B23] Ma X. Jiang G. Fu S. Fang J. Wu Y. Liu M. (2018). Enhanced network efficiency of functional brain networks in primary insomnia patients. *Front. Psychiatry* 9:46. 10.3389/fpsyt.2018.00046 29515469 PMC5826384

[B24] Marcos-Vidal L. Martínez-García M. Pretus C. Garcia-Garcia D. Martínez K. Janssen J. (2018). Local functional connectivity suggests functional immaturity in children with attention-deficit/hyperactivity disorder. *Hum. Brain Mapp.* 39 2442–2454. 10.1002/hbm.24013 29473262 PMC6866394

[B25] Maslov S. Sneppen K. (2002). Specificity and stability in topology of protein networks. *Science* 296 910–913. 10.1126/science.1065103 11988575

[B26] Menon V. (2013). Developmental pathways to functional brain networks: Emerging principles. *Trends Cogn. Sci.* 17 627–640. 10.1016/j.tics.2013.09.015 24183779

[B27] Miao B. Zhang Y. (2017). “A feature selection method for classification of ADHD,” in *2017 4th International Conference on Information, Cybernetics and Computational Social Systems (ICCSS)*, (Piscataway: IEEE). 10.1109/ICCSS.2017.8091376

[B28] Miao B. Zhang L. Guan J. Meng Q. Zhang Y. (2019). Classification of ADHD individuals and neurotypicals using reliable RELIEF: A resting-state study. *IEEE Access* 7 62163–62171. 10.1109/ACCESS.2019.2915988

[B29] Milo R. Shen-Orr S. Itzkovitz S. Kashtan N. Chklovskii D. Alon U. (2002). Network motifs: Simple building blocks of complex networks. *Science* 298 824–827. 10.1126/science.298.5594.824 12399590

[B30] Nichols T. E. Das S. Eickhoff S. B. Evans A. C. Glatard T. Hanke M. (2017). Best practices in data analysis and sharing in neuroimaging using MRI. *Nat. Neurosci.* 20 299–303. 10.1038/nn.4500 28230846 PMC5685169

[B31] Pereira-Sanchez V. Franco A. R. Vieira D. de Castro-Manglano P. Soutullo C. Milham M. P. (2021). Systematic review: Medication effects on brain intrinsic functional connectivity in patients with attention-deficit/hyperactivity disorder. *J. Am. Acad. Child Adolesc. Psychiatry* 60 222–235. 10.1016/j.jaac.2020.10.013 33137412

[B32] Power J. D. Barnes K. A. Snyder A. Z. Schlaggar B. L. Petersen S. E. (2012). Spurious but systematic correlations in functional connectivity MRI networks arise from subject motion. *Neuroimage* 59 2142–2154. 10.1016/j.neuroimage.2011.10.018 22019881 PMC3254728

[B33] Pulini A. A. Kerr W. T. Loo S. K. Lenartowicz A. (2019). Classification accuracy of neuroimaging biomarkers in attention-deficit/hyperactivity disorder: Effects of sample size and circular analysis. *Biol. Psychiatry Cogn. Neurosci. Neuroimaging* 4 108–120. 10.1016/j.bpsc.2018.06.003 30064848 PMC6310118

[B34] Rubinov M. Sporns O. (2010). Complex network measures of brain connectivity: Uses and interpretations. *Neuroimage* 52 1059–1069. 10.1016/j.neuroimage.2009.10.003 19819337

[B35] Sanchez-Alonso S. Rosenberg M. D. Aslin R. N. (2021). Functional connectivity patterns predict naturalistic viewing versus rest across development. *NeuroImage* 229:117630. 10.1016/j.neuroimage.2020.117630 33401011 PMC12021493

[B36] Schmahmann J. D. (2019). The cerebellum and cognition. *Neurosci. Lett.* 688 62–75. 10.1016/j.neulet.2018.07.005 29997061

[B37] Shao L. You Y. Du H. Fu D. (2020). Classification of ADHD with fMRI data and multi-objective optimization. *Comput. Methods Prog. Biomed.* 196:105676. 10.1016/j.cmpb.2020.105676 32791440

[B38] Shaw P. Eckstrand K. Sharp W. Blumenthal J. Lerch J. Greenstein D. (2007). Attention-deficit/hyperactivity disorder is characterized by a delay in cortical maturation. *Proc. Natl. Acad. Sci. U.S.A.* 104 19649–19654. 10.1073/pnas.0707741104 18024590 PMC2148343

[B39] Stam C. J. (2014). Modern network science of neurological disorders. *Nat. Rev. Neurosci.* 15 683–695. 25186238 10.1038/nrn3801

[B40] Tao J. Jiang X. Wang X. Liu H. Qian A. Yang C. (2017). Disrupted control-related functional brain networks in drug-naive children with attention-deficit/hyperactivity disorder. *Front. Psychiatry* 8:246. 10.3389/fpsyt.2017.00246 29209238 PMC5702526

[B41] Wang J. Wang X. Xia M. Liao X. Evans A. He Y. (2015). GRETNA: A graph theoretical network analysis toolbox for imaging connectomics. *Front. Hum. Neurosci.* 9:386. 10.3389/fnhum.2015.00386 26175682 PMC4485071

[B42] Wang L. Zhu C. He Y. Zang Y. Cao Q. Zhang H. (2009). Altered small-world brain functional networks in children with attention-deficit/hyperactivity disorder. *Hum. Brain Mapp.* 30 638–649. 10.1002/hbm.20530 18219621 PMC6870909

[B43] Wang Y. Tao F. Zuo C. Kanji M. Hu M. Wang D. (2019). Disrupted resting frontal–parietal attention network topology is associated with a clinical measure in children with attention-deficit/hyperactivity disorder. *Front. Psychiatry* 10:300. 10.3389/fpsyt.2019.00300 31156474 PMC6530394

[B44] Watts D. J. Strogatz S. H. (1998). “Collective dynamics of ‘small-world’networks. *Nature* 393 440–442. 10.1038/30918 9623998

[B45] Xia S. Foxe J. J. Sroubek A. E. Branch C. Li X. (2014). Topological organization of the “small-world” visual attention network in children with attention deficit/hyperactivity disorder (ADHD). *Front. Hum. Neurosci.* 8:162. 10.3389/fnhum.2014.00162 24688465 PMC3960496

